# Objectivity interrogation of racial scholarship in psychology and management

**DOI:** 10.1038/s41598-024-63236-z

**Published:** 2024-05-31

**Authors:** Brittany Torrez, Cydney H. Dupree, Michael W. Kraus

**Affiliations:** 1 Yale University, New Haven, USA; 2 University College London, London, UK; 3 Northwestern University, Evanston, USA

**Keywords:** Racism, Objectivity, Academia, Self-presentation, Psychology, Human behaviour, Psychology

## Abstract

Scholars of color remain underrepresented in US institutions in academia. In this paper, we will examine one factor that contributes to their continued marginalization in psychology and management: the scientific method’s commitment to traditional notions of objectivity. We argue that objectivity—defined as practices and policies rooted in the heightened value placed on a research process that is ostensibly free from bias—is central to the prominence of primarily White scholarship in psychology and management research and remains central to knowledge production. To investigate this, we employ a mixed-methods approach, integrating qualitative and quantitative data to codify how scholars of color experience *objectivity interrogation*s, or written and verbal questioning in academic contexts that implicates their scientific rigor. We also identify how scholars of color engage in *objectivity armoring*, or self-presentational strategies (toning down and stepping up) to contend with these interrogations. Finally, we reveal these toning down processes in language use within publications on racial scholarship. Overall, these studies reveal the unique challenges scholars of color face to legitimize and validate their work on race and racism within predominantly White institutions and disciplines.

## Introduction

In US social science research across time, scholars of color have consistently found themselves forced to the margins^1–3^, and recent analyses indicate that these conditions continue to describe the experiences of scholars of color in academia^4^. According to separate scoping reviews of the literature in psychology and management over four decades, scholarship in psychology is largely dominated by White scholars^4,5^. These conditions highlight a status quo where knowledge in psychology and management is generated by, for, and about White people.

In this study, our goal is to better understand this process of racial marginalization in US psychology and management racial scholarship—which we define as research that is conducted by, for, and about people of color. We accomplish this through a combination of qualitative and quantitative methodology that attempts to (a) understand researcher experiences of producing racial scholarship in their own words, and (b) assess the end products of that racial scholarship in the form of published academic writing.

There are many structural factors that force scholars of color to the academic margins; these have been covered elsewhere^6,7^. Our investigation led us to one specific, salient challenge to scholars of color conducting racial scholarship in the US that relates to commitments, in some social sciences, to objectivity in the context of research epistemology. Specifically, academic knowledge production in psychology and management research is judged by objectivity norms—whether the research is methodologically rigorous, neutral, and detached from personal interests^8^. Here, we argue that racial scholarship produced by scholars of color, in particular—which tends to center the unique perspectives of the racially marginalized and challenge the (predominantly White) power structures—is perceived as running counter to these objectivity norms. In mainstream academic spaces, racial scholarship conducted by scholars of color, therefore, risks perception as less credible, less legitimate, and more biased than scholarship that does not center marginalized perspectives and experiences.

Objectivity, defined as the “extent to which a researcher’s methods are free from prejudice”, is typically upheld across some areas of social science as a norm indicating scientific rigor, personal detachment, and neutrality^1,8^. These norms of objectivity are distinctly positivist. Positivism asserts that all truth is verifiable and that scientific evidence exactly reflects the reality of the world—completely free of values^9^. Thus, a social scientific positivist perspective values approaching research from a detached perspective that is free from prejudgment; indeed, some social science research manuals highlight the importance of objectivity^10^.

As social scientists, we value skepticism about our own and others’ research. However, no pure social science is colorblind or otherwise abstracted from context^11^. Nevertheless, fields like psychology and management, in the US and elsewhere, subscribe to forms of positivist epistemology^12^. Positivist epistemology, because it is similar to philosophies of science in the natural sciences, has historically enhanced the legitimacy of social sciences through shared methods and practices^13–15^. Thus, scholarship in psychology and management has tended toward a focus on objectivity and positivism^10,16^.

Racial scholarship (and indeed, any scholarship that derives from marginalized identities) is inconsistent with positivist epistemology for several reasons^17,18^. First, as stated above, because people’s racioethnic identities and associated contexts shape perception and judgment there is no pure form of social science that can be fully colorblind or wholly extracted from its context^10^. Second, racial scholarship often rejects principles of neutrality, and instead, directly or indirectly highlights existing racialized power structures. Norms of detachment and objectivity have a history in some US social science^19^, nevertheless, many scholars of color have been, and continue to be, interested in social sciences as a means through which they can illuminate (and clarify) the unique experiences and insights offered by people of color^20^. Third, racial scholarship in the social sciences is often informed by alternative epistemologies. For example, Black feminist epistemology asserts that as each group of oppressed peoples furthers the ideas offered by their unique standpoint this builds toward greater truth and knowledge^20^. Black feminist thought challenges traditional notions of what objective truths are, as well the existing systems and structures of power that allows one to arrive at them^20^.

The widespread embrace of positivism has left psychology and management, at best, ignorant of (and at worst, dismissive of) the notion that scholars’ identities, values, and context influence truth—a central assertion of racial scholarship. As scholars of color produce racial scholarship in psychology and management, their research is invariably informed by their unique racioethnic identities and alternative epistemological perspectives. This suggests that scholars of color producing racial scholarship are likely to contend with *objectivity interrogations* that arise from positivist norms.

Social scientists are likely to encounter objectivity norms at all phases of the research process, from idea generation to promotion and tenure review. To achieve traditional forms of legitimacy (e.g., faculty positions and publications) along with their material benefits, US social scientists must adopt strategies for demonstrating objectivity. While all scholars face questions about research rigor, methodological expertise, and quality assurance checks, scholars of color conducting racial scholarship are particularly likely to face objectivity interrogations for the reasons articulated above. We therefore expect scholars of color conducting racial scholarship to engage in self-presentational strategies, which we refer to as *objectivity armoring*, to manage these norms.

To avoid objectivity interrogation, scholars of color may reduce authentic expression of their racial scholarship—for example, downplaying their connections to communities impacted by equity-enhancing policies. Such expression could implicate one’s objectivity by suggesting that their scholarship is driven by personal investment. Much like how racial minorities downplay their stigmatized racial identities in the workplace^21,22^, scholars of color studying race may disassociate themselves from objectivity interrogations by attempting to present their research in ways that lends itself to perceptions of enhanced objectivity. This self-presentational approach may also include managing the potential discomfort of White audiences as they learn about racial scholarship, increasing perception of conventional standards of rigor through additional training and quantification, and avoiding discussions of racialized power structures or racial scholarship entirely. We examine these patterns of self-presentation in our research.

To examine objectivity interrogation and armoring, we employed a multi-method approach that integrates qualitative and quantitative methods, including in-depth interviews and archival analyses. We first used interviews with psychology and management scholars studying racial issues to examine the production of racial scholarship—and the role of objectivity in that process. Although a quantitative investigation of this phenomenon is possible (and has been conducted in other domains^23^), such designs do not allow for an in-depth understanding through firsthand accounts of scholars’ lived experiences^4,24^. Qualitative interviews allow us to directly attend to these experiences.

Interviews focused on scholars’ experiences communicating racial scholarship during presentations: in lab meetings, with individual faculty or peers, or at large conferences. Social science research is normatively subject to public scrutiny during research presentations. Such presentations also allow participants to engage in a wide variety of self-presentational techniques and are an important site of evaluation in academia^25^. Thus, examining research presentations can help us understand the role of objectivity in evaluative contexts shared by psychology and management scholars across the US. Importantly, we recruited scholars from multiple racioethnic groups, as we expected this phenomenon may depend on scholar race.

Our interviews were designed to reveal self-presentational techniques that scholars of color employ to influence perceptions of objectivity—e.g., toning down authentic expression of their identities. However, we reasoned that these presentation strategies could shape the publication process, with implications for how scholars represent their racial scholarship within journal publications. We therefore explored this possibility in an archival analysis of published scientific abstracts.

Our archival analysis investigated two possible outcomes of objectivity interrogation that were related to the self-presentational strategies cited in interviews. The first relates to discussions of power and race. One category of insight from racial scholarship is that it can clarify and reveal racialized power structures that explain how rights, resources, and privileges are racially patterned^7^. Scholars of color who are concerned about appearing objective may constrain their word use in racial scholarship, limiting language that highlights these power dynamics. White scholars, by contrast, may not experience the same pressures to dilute discussions of power. A second self-presentational response to objectivity interrogation is using more positive and less negative language. Scholars of color whose racial scholarship is interrogated may use more positive and less negative language to reduce White reviewers’ and editors’ potential discomfort. This may improve the publication process for scholars of color conducting racial scholarship, but it may have a cost of requiring these scholars to write about topics in a more positive, but potentially less authentic manner.

We test these two possible consequences of objectivity interrogation with an archival analysis of racial scholarship in psychology and management journals over more than 70 years. Through linguistic text analysis^26^, we examined whether scholars of color and White scholars exhibited different language related to power and emotion in racial scholarship.

## Results: scholar interviews

### Objectivity interrogations of scholars of color

A summary of our coding scheme and additional representative quotations appear in Table 1. Additional examples of each category of interrogation and armoring can be found in the supplementary materials. A majority of scholars of color (87 percent) shared at least one experience of objectivity interrogation. In this section, we will present our findings regarding the types of interrogations scholars encountered. Responses fell into two conceptual categories: *disbelief* and *pushback*.

**Table 1:** Thematic coding analysis of objectivity interrogations and armoring.

Themes	Codes	Prevalence (%)	Representative quotes
Objectivity interrogations			
Disbelief	Disbelief	20	“I’ll say a fact and…the response is, ‘I just find that hard to believe.’ I’m like, ‘Okay, well … I’m telling you a fact’.”
Pushback	Ideological Pushback	61	“White, tenured faculty would come out and say things like, ‘Oh yeah, all those people studying their own issues,’ when they were talking about people studying diversity.”
	Methodological Pushback	43	“One of my other lab mates, she would present qualitative work and they would come at her very critical, in a very non-constructive manner, which didn’t make sense because none of them did qualitative work. … I’m like, ‘No, that’s not how qualitative methodology works.’ ”
Objectivity armoring			
Toning down	Constraint	53	“Some of my more radical beliefs… they’re supported by what I’m saying, but I don’t lay it out that thickly.”
	Avoidance	43	“I actually really think [switching topics] helps. So sometimes I’m talking about a category that’s not threatening to the people in the room, and sometimes I’m talking about a category that is threatening.”
Stepping up	Overpreparation	55	“It helps me think ahead of time, what are the questions that I’m going to get? How can I frame this in a way that very clearly communicates the scope of my idea and the boundaries of my idea?”
	Quantification	25	“Folks tend to undermine the methods that people of color use or assume that they don’t know how to do certain things. I take extra care to learn those things and make sure that it’s extra clean, and make sure I always check for small errors and stuff like that. Because, I’d feel we’re more likely to get critiqued on the little things.”
	Exacting communication	20	“It’s usually me spending hours … just going through the slides and getting down the wording exactly as I want it, and the intonations and inflections and my hand movements and like the dramatic pauses and all of that. So I practice that like a play. And so that’s what prepares me.”

All percentages and quotations are from racial minority scholars.

#### Disbelief

Disbelief fundamentally called into question a researcher’s judgment by directly questioning whether a racialized phenomenon was true or real, casting doubt on the researcher’s ability and judgment. This kind of interrogation is in line with our assertion that research that challenges normative standards (e.g., research revealing the racialized nature of society) may prompt doubt in researcher judgment, eliciting a more skeptical review process. The denial of racism is a common response employed by people who, intentionally or otherwise, want to think of society as meritocratic or egalitarian^27,28^. Indeed, this reaction to the experiences of marginalized groups has costs for scholars of color studying racial scholarship. Disbelief introduces systematic doubt in a program of research (and the researcher’s judgment and ability) as soon as the researcher’s identity and topic are known.

Disbelief in basic, easily-corroborated, claims by scholars of color conducting racial scholarship is difficult to reconcile with academic norms of knowledge production. For instance, bias, even explicit bias, persists today^29,30^, and yet, one scholar of color shared how their work on bias was interrogated for its veracity: “One of the kind of strongest themes of my early talk was probably the question of, ‘Do people even say biased things?’ and, ‘Is that comment in your study even biased?’” (RM_104). This scholar described senior graduate students asking them, “Why does this matter?”. In response they internalized, “I have no idea. Everything I think is terrible.” Disbelief was raised about a basic experience that scholars of color face—one that has decades of evidence in the social sciences^31^. The scholar’s response was to internally question their own judgment and capacity to conduct research.

Another interviewee recalled a moment at a scholarship panel on stereotyping and discrimination when someone interrupted to say that they did not believe stereotyping was “real”:

There’s only been very few moments in my life where even as a person, not as much as a scholar, where I feel like my personhood was questioned that explicitly to my face. I think such a large part of being a person of color and ethnic minority, or gender minority means that you have experienced stereotyping, discrimination, and bias all the time in a thousand different ways. To have someone say that’s not real, was just to basically say like, ‘Oh, your lived experience is just not real, you’re making it all up’, which people hear all the time when they get gaslit. (RM_13)

Here the scholar highlights the connection between their “personhood” and their racial scholarship. Scholars of color conduct racial scholarship that is often novel to social sciences because it comes from marginalized lived experiences. Disbelief cast at these experiences and the resultant research is an explicit and public questioning of a marginalized scholar’s judgment. Disbelief can also convert to explicitly-stated concerns about a marginalized scholar’s judgment and even their fit in the field. On the most extreme end, disbelief can be based in ideologies of White superiority:

I had done a project on the disproportionate rate of Black males in special education programs … and this White woman came up to me and the first thing she said to me was, ‘You don’t think that there’s more Black men in special education because they’re just not as smart?’ (RM_26)

#### Pushback

Interrogations—defined here as pushback—reinforce the theorized skeptical review process. The vast majority of scholars of color recalled an experience of pushback when sharing their research. Here, we categorize pushback into two first-order categories: (1) *ideological pushback*, implicating the scholars’ positionality in relation to an ideological agenda, and (2) *methodological pushback*, implicating the scholars’ methodological and analytical choices.

##### Ideological pushback

Although both types of pushback were frequent in interviewees’ responses, ideological pushback was more common, with more than three quarters of scholars of color reporting an experience of ideological pushback. As with disbelief, these experiences ranged from more explicit (e.g., angry statements directed at scholars) to more subtle (e.g., suggesting that the research has a political agenda). One faculty member shared more explicit examples, in which they were asked to mind how their racial identity influences their research:

I have had people ask me, ‘Well, you’re doing this research because you as an [racial minority identity redacted] are of course curious about this.’ And then the second line following that is typically, ‘But you want to make sure you don’t inject your own prejudices into your research.’ Which then I say, ‘Yes, I understand that but we have to do research that fascinates us.’… I guess the underlying motivation for what people are trying to tell me is always keep your biases in check. (RM_102)

Although the research topic was novel and innovative, it was reduced to “me-search”. The scholar’s racial identity calls into question their ability to “keep [their] biases in check” (RM_102). This narrative highlights the implications of interrogation for the perceived rigor of the work. Other scholars of color noted that their work on diversity is often perceived as “lower in quality or political and agenda-driven” (RM_12) and that “if I’m studying [racial minorities] and I am a [racial minority], then I automatically have an agenda” (RM_14). Others noted that their work is not perceived as “legitimate” (RM_19) and is seen as “more of an opinion than scientific” (RM_8). One scholar noted that the stigma against theorizing from lived experiences was unique to racial scholarship:

When people bring in their personal selves or stories about how they got to the research question that’s not around race or inequality, it’s seen as kind of, ‘Wow you are really observant of your surroundings and you have a great way to observe different social phenomenon in the real world,’ but as a person of color having experiences it’s kind of like, ‘Of course you’re going to think that.’ (RM_11)

We note how far-reaching this ideological pushback is. Although we expected pushback to be less likely for more senior scholars, we did not observe such patterns. Students and faculty alike shared experiences where their scholarship was minimized, discounted, or discredited due to perceived ideologies—even if these ideologies could not be named.

##### Methodological pushback

Over half of interviewees of color shared experiences with methodological pushback. Such interrogation primarily discredited or devalued one’s methodological choices. Scholars studying racial minorities were often critiqued for lacking a White control group—implying that one can only understand racial minority experiences via comparison with White experiences^32^. One scholar recalled, “We had this norm that you need a White control condition like you need to compare… That is the norm. And it was frustrating because it’s like, ‘Do we need that to just talk about minority experiences?’” (RM_16). Scholars were asked by reviewers to explain how they can “generalize”, even though “When we have majority White samples, no one asks that question, it is just seen as a standard.” (RM_19).

However, any methodological choice could be called into question—including research design, method of data collection, and/or analytical techniques. One scholar described how their recruitment method was interrogated by a reviewer:

Someone was like, ‘Oh snowball sampling is never a good methodology.’ And I was like, ‘Actually it’s a good methodology if you want to get a certain demographic group that is hard to access … that’s the whole point of snowball sampling.’ And they were like, ‘Well yeah, it’s only good if you want to get a very, very, very specific sample.’ Like an ethnic group is not specific enough. (RM_13)

Snowball sampling is one of the most empirically-effective strategies to recruit minoritized populations^33^. However, this methodology was called into question in the context of racial scholarship. A scholar’s statistical reporting techniques may also be interrogated to reject ordinary claims about racism (e.g., that White people may be racist):

He’s just like, ‘You know, your effects are really, really small. That’s only some White people. You really need to show on your figures that this is a small percentage of White people who act this way.’ (RM_7)

Overall, scholars of color conducting racial scholarship were often subject to a variety of interrogations that called into question their objectivity and credibility as scholars. Sometimes these were more subtle (e.g., challenging research findings based on effect sizes), and other times they were more explicit (e.g., implying that the predominance of Black men in special education is due to their inherent lack of intelligence).

### Objectivity interrogation among white scholars and on non-racial scholarship

The above data provide evidence of objectivity interrogations levied against scholars of color studying racial issues. However, we wondered whether this phenomenon was a feature of psychology and management presentations more broadly or if it was wielded disproportionately at racial scholarship (or even more specifically, at racial scholarship conducted by scholars of color).

Scholars of color reported greater pushback when presenting scholarship centering race than when presenting work where race is not central. For example, a male tenured professor described the relative difficulty of talking about race versus gender—a non-marginalized identity for them—in the classroom, saying, “People trust that what I’m saying about gender is more honest because it goes against my self-interest … When I’m talking about race, I think there’s this, ‘Well, what’s in it for him?’ There’s that kind of feel to it.” (RM_20). As theorized, the perceived lack of objectivity makes it harder for scholars of color to present scholarship on race. This scholar further described the frustration that comes from knowing that racial scholarship could be more effectively delivered by their White colleagues; unfortunately, these colleagues did not want to deliver such content.

[White colleagues] can really go a long way towards carrying the message … yet they’re the ones that don’t want to engage. They don’t want to talk about implicit attitudes. They don’t want to talk about racism. They’re just uncomfortable. And I’m just like, ‘I don’t understand. I have to teach like the psychology of motivation. I don’t study that, but I learned it and I talk about it. Why can’t you all just learn this as a research topic and teach it?’ (RM_20)

A common thread that scholars of color noted was that the anxiety that pervades presentations on racial scholarship is lessened during non-race-related presentations. Another scholar described the anxiety induced by presenting racial scholarship, in particular, even foregoing a job talk in order to avoid presenting on this topic:

It was really the topic of talking about racism in front of academics, that was nerve wracking to me. To the point I remember I almost felt sick when I was about to give the talk. And that’s one of the reasons why I only got two job talk offers … I turned down the other [job talk], because I had a hesitancy to go around and talk about this topic in the job market. (RM_19)

White scholars, regardless of what they studied, were much less likely to report experiencing interrogations of their work on racial scholarship (see supplementary materials). These scholar interviews suggest frequent and widespread disbelief and pushback around racial scholarship specifically directed at scholars of color. Moreover, this objectivity interrogation was less common and less intense for scholars of color studying topics not explicitly racialized and observed to be less frequent and intense by White scholars studying racial scholarship. We next detail self-presentational strategies that scholars of color engage in to manage objectivity interrogation.

### Self-presentational techniques: objectivity armoring

Several self-presentational techniques to enhance perceptions of objectivity (i.e., objectivity armoring) were described by scholars of color conducting racial scholarship. Objectivity armoring fell into two conceptual categories, which we describe as *toning down* and *stepping up*.

#### Toning down

One common technique described was toning down self-presentation: softening findings, employing euphemisms, and avoiding certain claims to align with academic audiences. We categorize toning down into two first-order categories: (1) *constraint* and (2) *avoidance*.

##### Constraint

Scholars of color often reported softening their communication to avoid potential threat or discomfort. We use the word constraint to implicate the power dynamics involved in this process. These scholars of color felt they had to limit or restrict their preferred way of communicating their research (typically, to be heard by White evaluators). This constraint involved a variety of techniques, which we delineate below.

I’ve figured out ways to talk about race in ways that are sort of generic sounding enough to get people to buy in. And then I start talking about what I’m talking about. So I found that talking more generally about group-based hierarchies abstractly is a way of getting people to accept that this stuff matters. … [it’s] a way of gently leading people in. (RM_20)

Scholars often found replacements for words that may be seen as threatening: “So instead of saying ‘modern racism’ all the time, you can switch it to say ‘bias’ sometimes, or ‘prejudice’ sometimes. Because if you are constantly repeating ‘racism, racism’, that can make people uneasy.” (RM_19). Several scholars of color described adjusting their nonverbal behavior (e.g., smiling) to soften the delivery of racial scholarship: “In these spaces we always have to wear a mask, right? So you know, you do that sort of like bemused professional smile.” (RM_7).

Scholars of color described constraint as a self-presentational strategy to disarm potentially-threatened audiences, achieve professional status (e.g., employment), and appease White audiences: “I think there are probably some more radical takes that I could put on that would get you in more trouble in the field, which I don’t do, because I don’t have a job yet” (RM_10), and “But those aren’t things you can just say during an interview. You don’t want that to limit your options.” (RM_27). Most commonly, scholars were explicitly taught these strategies by their advisors or mentors, often in service of gaining employment.

People were like, ‘you’re making everyone in your audience feel like you’re calling them a racist … You can’t navigate that well enough to avoid making them feel like they’re biased .., and you’ve got to get a job.’ (RM_104)

##### Avoidance

Although scholars of color often tried self-presentational constraint to appear more objective, they sometimes felt the only option was to abandon racial scholarship projects altogether, often in favor of research that will be more interesting to White advisors and audiences.

I want to be known as a [race] scholar. … But one thing that I’ve struggled with is I’ve thought about almost all of this by myself. Whereas with all this other stuff that I’m doing I have several people who are willing to talk through my findings and really try to figure out what’s going on. Whereas for my [race] stuff, it really has felt, it’s just me out here on a whim not knowing what the fuck I’m doing. And so I, after that experience, I definitely think I shifted to non-[race] stuff. (RM_4)

As with the example above, avoidance was often imposed by colleagues and advisors who were unwilling or unable to engage with race-related research topics: “I just was like, ‘If I stick with social class, then he’ll feel like it’s something he can relate to. … That’s safe for both of us.’ And so I just kind of stayed in my lane and did that.” (RM_21). Because of this, scholars of color felt constrained in the topics they were able to study.

#### Stepping up

The first two self-presentational strategies (constraint and avoidance) involve scholars of color toning down their manner of speaking or approach to research. Another strategy involved scholars “stepping up” their communication techniques to avoid interrogation. Stepping up was comprised of three categories: (1) *overpreparation*, (2) *quantification*, and (3) *exacting communication*.

##### Overpreparation

Overpreparation involved scholars spending significant time preparing specific details for their presentations, anticipating interrogations and rehearsing responses, and accumulating evidence to avoid misunderstanding or appearing incompetent. This resulted in near constant adjustment: “I’ll go to visit people’s labs and present it, meet people at coffee shops, get on Skype with people and just take notes and keep adjusting and keep adjusting” (RM_18). This approach was described as cognitively depleting:

You do a lot of legwork on the backend of knowing everything in and out. … Which is a waste of mental things … When you’re dealing with that sort of environment, you just want to be prepared on everything. I like tend to over prepare, which is good. I think people tend to think that I present well for that reason, but I think it’s unnecessary at a certain point. (RM_13)

Like toning down practices, this stepping up approach is often informed by scholars’ perceptions that their scholarly integrity and objectivity are in question due to their racial identity:

Whether people view me as objective is implicated because of my identity. And, so, that’s also why I like to know my talk back and forth. I need to have thought about all the different questions that I could get and how I’m going to answer them. (RM_17)

Another noted that scholars were aware that they can be perceived as biased, and this motivated overpreparation:

By being a [racial identity redacted], I’m usually the only one in the room sometimes, when I’m presenting my work… Our anticipation of people believing that we’re biased affects our presentations, so we want to make sure every fact, every decimal point is in the right place … I have to do my due diligence in my work, because it’s already, in my mind, being seen as coming through a biased lens. (RM_14)

Overpreparation often involved monopolizing a large amount of talk time to “prove” certain points to a predominantly White academic audience:

The strongest themes of my early talks were probably the questions of, ‘Do people even say biased things?’ and, ‘Is that comment in your study even biased?’ So I would spend tons of time having to prove those two points. (RM_104)

In fact, scholars of color often described being interrupted to explain racialized topics that were quite germane yet not well understood by their audiences. For example, another scholar lamented having to spend time explaining the basic concept of intersectionality, “whereas a lot of my other peers were talking about all of the studies that they did” (RM_4).

##### Quantification

Scholars of color seem to understand that positivism is dominant in their discipline and employ quantitative methodologies to increase their perceived objectivity (and therefore, credibility). Such quantification included scholars amplifying their statistical techniques, analytic approaches, or studies to align with methodological standards of objectivity:

More on the statistical end and really just trying to read up on, for example, a structural equation model … thinking about all the little things, knowing that I can explain everything in my syntax and everything in my output … I’m always doing that, and going through my scripts and making sure that they’re all replicable and close to them and people can know what I did. Just taking those steps. (RM_22)

This scholar specifically tied quantification to their racial identity, noting:

[People of color] have to be careful about just the methods they use, and how they use them in terms of making sure you’re using the most rigorous techniques available for whatever method it is… Making sure you’re trying to live up to the best practices, whatever method that is. (RM_22)

Scholars who used quantification strategies felt more confident in their work: “It’s hard to argue with numbers. If I were to point something out that is uncomfortable, especially for the majority group, well, I don’t know what to tell you guys, that’s what the numbers told me.” (RM_103). Moreso, one scholar mentioned that because they “have been developing rigorous methods to quantify these things” (RM_12), their work was received more positively.

##### Exacting communication

Finally, scholars also employed exacting communication: a method of speaking in which they carefully and meticulously selected their verbiage for a presentation. This strict presentation strategy is closely related to the toning down practice of constraint; to avoid having their work misunderstood or rejected, scholars spent time selecting precise words or phrases to present their research. For example, one scholar mentioned they were “very careful about the claims that I was making about the research” (RM_25), while another said, “I don’t want my words misconstrued or misinterpreted. I like to be very clear and exact with what I mean” (RM_11).

As with the previous self-presentational strategies, this technique surfaced when presenting to predominantly White audiences, largely to avoid their own and audience discomfort:

[When] I am the only person of color in the room, you know, they can’t help but make things feel a little uncomfortable, at least for me. Or make me think twice sometimes about exactly what I say, or how I phrase it. (RM_8)

### Objectivity armoring among white scholars

We sought to better understand the boundaries of the self-presentational strategies employed by scholars of color conducting racial scholarship by examining White scholars self-presentational strategies. In our interviews, we observed less exacting communication patterns employed by White scholars. Moreover, when White scholars did report objectivity interogations, they were not described as particularly concerning. See the supplementary materials for representative quotes on this topic.

Our interviews with scholars of color conducting racial scholarship revealed that these scholars experienced disbelief and pushback around their research—interrogations that questioned basic facts and their ideological commitments, surfacing broader concerns about their own bias on racial scholarship. To avoid interrogation, these scholars toned down their language and presentation styles and stepped up their quantification and communication patterns. Scholars of color mentioned having fewer of these objectivity interrogation experiences in scholarship that was not explicitly racialized. Moreover, White scholars observed rarer instances of objectivity interrogation, and thus reported using fewer self-presentational strategies to contend with these interrogations.

## Results: archival analyses of racial scholarship

We conducted text analyses of racial scholarship, testing for linguistic differences in abstracts authored by scholars of color (coded “1”) versus those authored by White scholars (coded “-1”). Three analyses tested differences in power and emotion language. We first treated lead author race as a between-subjects factor. Next, we used a linear regression analysis where we added control variables for abstract word count, journal impact factor, author h-index (a correlate of author status), and publication year. Lastly, we used a linear mixed-effects model accounting for abstracts nested within journals with the same control variables. Results were consistent across analyses.

In the between subjects analysis, lead authors of color wrote abstracts with significantly fewer power words (*M* = 3.07) than White authors (*M* = 3.56; *t*(1686) = 3.243, *p* = 0.001) and significantly more positive emotion words (*M* = 2.50) than White authors (*M* = 2.17; *t*(1686) = -3.219, *p* = 0.001). There were no effects on negative emotion words *t*(1686) = 0.153, *p* = 0.879. This analysis was also consistent with an analysis, reported in the supplementary materials, controlling for special cases where lead authors appeared more than once in our data.

In the linear regression analysis, lead authors of color again wrote abstracts with significantly fewer power words *B* = − 0.102, *t*(1191) = − 3.497, *p* < 0.001 and significantly more positive emotion words than White authors *B* = 0.101*, t*(1191) = 3.485, *p* < 0.001. There were no differences in negative emotion words *B* = − 0.031, *t*(1191) = -1.061, *p* = 0.289. Power words were more common for longer abstracts and for more recent publications whereas positive emotion words were more common from lead authors with higher h-indexes and more recent publications. See the supplementary materials for full model results.

In the linear mixed model analysis where abstracts were nested within journals, lead authors of color again wrote abstracts with significantly fewer power words *b* = − 0.226, *SE* = 0.093, *t*(1180.451) = − 2.433, *p* = 0.015 and significantly more positive emotion words than White authors *b* = 0.226, *SE* = 0.062, *t*(1082.186) = 3.667, *p* < 0.001. There were no differences in negative emotion words *b* = − 0.103, *SE* = 0.076, *t*(1173.303) = − 1.358, *p* = 0.175. Power words were more common for more recent publications whereas positive emotion words were more common from lead authors with higher h-indexes and more recent publications. See the supplementary materials for full model results.

Overall, these archival analyses provide initial linguistic evidence for the toning down strategies that scholars of color conducting racialized scholarship described in interviews. Across analyses, results indicate that scholars of color publishing racial scholarship tend to use less power-related and more positive language than White scholars.

## Discussion

Across one qualitative interview study and one quantitative archival analysis of published abstracts in psychology and management, we examined scholars’ experiences conducting scholarship related to race. Scholars of color report facing interrogations of their objectivity—interrogations that implicate their research as flawed on both ideological and methodological grounds. Our studies also reveal how scholars of color contend with these interrogations: In both our interview and archival analysis, scholars of color tended to use self-presentational strategies that make the work more palatable to (mostly White^4^) academic audiences, including toning down their communication. Scholars of color also reported employing higher standards of quantification and evidence that were, at times, specifically tied to expectations of interrogation of their objectivity. Overall, these studies reveal the additional challenges scholars of color face when conducting racial scholarship, challenges that their White counterparts largely did not report facing, and the implications for how scholars of color are socialized to discuss race and racism within academic contexts—by using less power-related and more positive language.

The fields of psychology and management are dominated by research with White participant samples, White authorship teams, and White editors and reviewers^4^. Our studies help articulate how this context shapes the experiences of scholars of color who are conducting research (on race and racism) that is counter normative to these conditions. The first contribution of this research is that it illuminates the experiences of scholars of color in their own words. The phenomenon of objectivity interrogation merits further empirical scrutiny with qualitative and quantitative methodologies, revealing the extent of objectivity interrogation and its costs on individual scholars, the social sciences, and our society^23,34^. Likewise, though the scholarship here is concerned with one form of marginalization based on racial oppression, theory and research indicate that other forms of marginalization (e.g., gender, disability status) shape perspectives on, and evaluations of, research. Future research could examine similar (and unique) costs and consequences of objectivity interrogation in these other domains.

This research can help us understand the potential costs of objectivity interrogation in relation to efforts to diversify academia. Given the extraordinary questioning that scholars of color report receiving, many scholars of color could have their careers delayed or derailed by this process. In the fields of psychology and management, PhD degree holders outnumber tenure track jobs by several orders of magnitude. If scholars of color receive additional scrutiny of their ideology and methodology when conducting racial scholarship, as we observed in these interviews, then these scholars must overcome additional barriers to meet standards for hiring, promotion, and tenure—barriers that are specific to objectivity interrogation.

This research also informs us about the costs of objectivity interrogation for scholars of color conducting racial scholarship even in the best-case scenario—when scholars of color persist in their research on race and racism and go onto successful careers, with many scholarly publications. Specifically, toning down practices ensure that scholars of color publish about race and racism with fewer mentions of power (e.g., abuse, control) and greater mentions of positivity (e.g., harmony, bonding), as our archival analysis suggests. These linguistic choices obscure the central role of power relations in the context of racism and hinder authentic expression for scholars of color.

Despite the numerous strengths of this research, there are several limitations that warrant further scrutiny. The research focuses on psychology and management scholarship on race and racism, and it does so from a US lens. This is a narrow context for understanding the dynamics of objectivity interrogation in the social sciences and limits our understanding of how these processes play out in other regions and cultural contexts. Though we cannot know how these processes surface in other contexts, from this investigation, it is generative to think about the specificity or generalizeability of objectivity interrogation.

The present work begins to illuminate how objectivity norms shape the evaluation of social scientists of color studying race. However, additional work is necessary to further clarify the process and consequences articulated here. This includes experimental work, already ongoing in other domains^23,34^. Qualitative work is also worth pursuing, particularly in social science disciplines and departments less dominated by positivism. Such work can help clarify how to conduct and support racial scholarship that minimizes the objectivity interrogations articulated in these scholar interviews.

Objectivity is a foundational tenet of the scientific process. Simultaneously, racism continues to pervade society^35^. Academia denies objectivity to scholarship that unequivocally names racialized power dynamics—this is inconsistent with reality, and even dangerous. We used scholar interviews and archival analysis to examine how objectivity norms disproportionately implicate racial scholarship conducted by scholars of color and thus influences these scholars’ self-presentation, ultimately shaping how race relations are discussed in academic work. Altogether, these findings have implications for the success of scholars of color conducting racial scholarship, as well as their capacity to sustain academic careers against pressures to align their scholarship with the status quo. Without careful attention to the downstream consequences of traditional objectivity norms, our adherence to its practices can reproduce the exact kinds of racial inequality social science scholars often seek to describe and remedy.

## Method: scholar interviews

The first author conducted 51 in-depth interviews with 31 scholars of color and 20 White scholars studying racial issues from September 2019 to May 2020, totaling approximately 36 exposure hours. All study methods for the scholar interviews and archival analysis were conducted according to Institutional Review Board guidelines at Yale University. Given the first author’s ethnic identity (Latina) and field of study (psychology and management) she was able to make use of personal networks in psychology (e.g., the Society of Personality and Social Psychology) and management (e.g., The PhD Project) to recruit potential participants. Scholars identified as studying racial issues were contacted via email and asked to complete a short intake survey, which included the consent form, demographic questions, and participants’ CVs (CVs were requested to review scholars’ research ahead of each interview). Once participants completed the survey, they were contacted via email to schedule an interview time. This sampling methodology is useful for contacting small communities, but one weakness is that such sampling introduces selection biases that make generalization to other groups less straightforward.

Approximately two-thirds were scholars in psychology and another third were scholars in management, which draws from psychology and related disciplines. Regarding career stage, 59% of participants were graduate students; other scholars’ positions represented a range of tenure in the field (e.g., post-doctoral scholars, untenured and tenured faculty). Approximately 69% percent of scholars identified as female; the rest identified as either male or non-binary. To protect the scholars’ identities, we will not provide their exact racioethnic breakdown. Instead, we refer to scholars as either a scholar of color (RM) or a White scholar (WH). This categorization structure also matches the themes that emerged from our scholar interviews.

Each interview began with a discussion of confidentiality, recording permissions, and a general discussion of the project background and interview goals. All scholars consented to participate in the study, which was approved by the Institutional Review Board at Yale University. Interviews followed a semi-structured interview guide broken down into three sections: (1) research interests, (2) experiences presenting research, and (3) how scholars’ racial identity shapes their experiences conducting and presenting racial scholarship. Interviews lasted between 30 and 50 min and took place over the phone. Notes and memos were taken throughout the interviews, and the audio recordings were transcribed verbatim, with identifiable information removed.

In the interviews, participants were initially told that we were interested in how researchers make sense of their research interests and their experiences sharing their work formally or informally. To gain a general sense of how participants framed their work, we first asked scholars to describe their research and its development. The interviewer then segued to a set of questions on presenting research, asking scholars how they felt people in their field (i.e., psychology or management) think about their research interests. Participants were then asked to describe a time when they presented their work. If necessary, the interviewer chose CV items to initiate conversation, picking one presentation on racial scholarship and one that did not reference race. After a general description of their experiences, the interviewer asked participants if they had ever encountered skepticism in response to their work, and, if so, to discuss it. Finally, informed by its spontaneous relevance in early interviews, we asked participants how they felt their racioethnic identity influenced their presentation experiences. Data collection continued until saturation was reached regarding participants’ experiences of objectivity interrogation and self-presentation^36^.

We used a form of content analysis for this study, drawing on analytic techniques derived from grounded theory^37^. In this form of analysis, the theory is generated directly from the data itself^38,39^. This bottom-up process is an inductive method, which allows the researchers to systematically comb through participants’ rich, detailed experiences. Themes emerge from these data, resulting in a nuanced but organized theory of objectivity interrogation and objectivity armoring that is derived directly from participants’ disclosed experiences in the field.

Following data collection, NVIVO qualitative data analysis software was used to document, track, organize, and summarize codes and themes. The first author then embarked upon a three-phase coding approach^40^. The first phase was open coding. The first author a) identified segments of text that refer to specific concepts, and b) allocated a certain code to that concept. This resulted in an extensive list of *codes* with which to label the data. The second phase followed the first two of Dey’s^40^ three aspects of qualitative data analysis: describing, classifying, and connecting. Initial reading of each text segment ensured comprehension (describing), followed by allocation of an initial code (classifying). All transcripts were coded, line-by-line, following this process.

The second phase followed the last of Dey’s^40^ three aspects of qualitative data analysis: connecting. Drawing on the full list of initial open codes developed in phase one, the first author examined the relationships between codes, sorting first-order codes representing similar ideas into second-order categories. In the final phase, we assessed categories for conceptual overlap and explored patterns that emerged. We then refined and connected the second-order categories, organizing them into main themes that could be interpreted^39,41^. Two theoretical dimensions emerged: (1) objectivity interrogations directed toward scholars; and (2) scholars’ self-presentational techniques. Throughout coding, we took care to examine the data for negative cases, paying particular attention to White scholars’ interrogative experiences and self-presentational techniques. We also interviewed scholars of color who do not study race. These cases will also be discussed and serve to enhance our understanding of the phenomena under investigation.

## Method: archival analysis

We first created an archive of scientific abstracts on racial scholarship by scraping key information from a total of 32 psychology and management journals on PubMed. This information included article titles, author names, abstract content, keywords, and publication dates (range: 1945–2021). We explicitly included 7 specialty journals focused on race or culture (e.g., *Journal of Black Psychology*) and a range of journals that varied in impact factors (*M* = 8.26, *SD* = 6.23, range: 1.11, 24.61).

We focused on articles about race-related topics. First, we developed a list of 169 race-related keywords by pulling keywords from the first 100 results after querying “race” on each journal’s website. We also included race-relevant keywords from prior research on racial scholarship^4^. Pulling articles that featured these keywords left us with 1,688 article observations from 26 journals (*n* = 621, specialty race journals; *n* = 1061, general journals).

We next determined the apparent racial identity of each article’s first author using two methods. We first used the *predictrace* package in R, which coded the authors as either White or scholars of color based on their names. Authors’ likely race was then confirmed manually by a team of research assistants, who sought additional information about the authors’ self-identified racial category and ethno-racial origins from their websites. This resulted in a total sample of racial scholarship manuscripts by 996 White lead authors and 692 lead authors of color. Analyses focus on lead author race because, though we cannot know with certainty whether lead authors wrote the abstract, APA guidelines state that lead authors make the most critical writing decisions within journal articles^42^. Analyses in the manuscript control for lead author h-index which is a proxy for citation impact (*M* = 32.59, *SD* = 27.46), journal impact factor (*M* = 5.01, *SD* = 3.987), abstract word count (*M* = 163.70, *SD* = 46.76), and publication year (*M* = 2010.60, *SD* = 7.40).

We conducted text analysis of the abstracts using Linguistic Inquiry and Word Count (LIWC) language analysis software^26^. We focused on power (e.g., authoritarian, abuse, control, elite, tyranny), positive emotion (e.g., friendly, happy, optimism), and negative emotion language (e.g., fear, depressed, disdain, hopeless) by drawing on LIWC-2022’s related dictionaries (e.g., Pennebaker et al., 2015). Analyses controlled for word count (*M* = 163.70, *SD* = 46.76). Words related to power (*M* = 3.41, *SD* = 2.89), positive affect (*M* = 2.27, *SD* = 1.99), and negative affect (*M* = 2.28, *SD* = 2.43) were all used by authors in their abstracts.

### Supplementary Information

Supplementary Information.

## Data Availability

Data generated for the archival study are available on the open science framework at https://osf.io/ardpy/. Full transcripts for scholar interviews are not available due to privacy concerns, but redacted versions can be accessed from the corresponding author based on reasonable request.
