# Supplementary material for: Objectivity interrogation of racial scholarship in psychology and management
Source: Sci Rep. 2024 May 31;14:12509. doi: 10.1038/s41598-024-63236-z (PMC11143200; doi:10.1038/s41598-024-63236-z)
Supplement: Supplementary file 1 — Supplementary Information. [file 41598_2024_63236_MOESM1_ESM.pdf]

Supplementary Materials

Results: Scholar Interviews

Additional examples of disbelief. The structure of psychology and management means that scholars of color have their racial scholarship evaluated by White audiences—audiences who do not have personal experiences of racial bias and discrimination. Disbelief is thus a feature of evaluation beyond research presentations and into the review process:

> "You have to get the reviewers to understand your paper, of course, and buy the ideas. But it's really hard when they don't have that perspective. There's no way they'll ever fully understand it, so it's how do you convince people of a reality that they've never lived? But that's part of the job and the research… convince people that this is really important when they've never encountered it—and never will." (RM_16)

Although these examples vary in the directness of their denial of established racialized phenomena (e.g., bias and stereotyping), questions of a scholar of color's judgment were sometimes made explicitly. For instance, some scholars reported being informed during presentations that their research was a poor fit for their discipline:

> "I got to the Q&A, and there were numerous questions, doubting the theory and whatever. Very, very little support. I don't recall anybody being like, this is a really great idea and I'm excited you're pursuing it. It was all very sort of negative and here are the problems … It got to a point where one of the faculty members outright said, 'I really just feel like this is not social psychology.'" (RM_23)

As highlighted above, while interrogations can emerge as explicitly negative commentary, they can also emerge as a lack of positive commentary, relative to departmental norms.

Additional examples of ideological pushback. On the more extreme end, a scholar described how, in response to their work on affirmative action, a White faculty member "flipped out" and felt "triggered" (RM_20). Importantly, the downstream consequences of such pushback can be severe, with one tenured faculty member noting how ideological pushback to their work will be permanently fixed in their tenure and promotion file:

> "At the time [of my review], it was represented that at least one person … questioned whether my research could ever be viewed as truly world-class science because of the 'ideologies behind them'… My review committee refused to take it out, and so it's ended up in my third-year review. It's still there. … The quality of my work has been questioned by people who see it as driven from ideology, that when questioned about what ideology they think I'm pushing, they will never say." (RM_104)

Additional examples of methodological pushback. Other aspects of the research design that may be called into question are the racial identities of the research assistants (RAs) conducting the studies:

> "[I was] walking through my methodology … The first question I got was about my RAs and the race of the RAs and how did the race of the RAs impact whatever? And I thought that was the most basic question; we obviously accounted for that." (RM_9)

Upon comparing the question-and-answer portion of their own and their classmates' first-year PhD talks, this scholar noted that the questions they received were decidedly "lower-tier"; the questions were not as carefully thought out, and by implication, did not constructively push the research forward.

Additional examples from non-racialized scholarship and from White scholars. Other examples of reduced anxiety around non-racialized scholarship were shared, researchers found it easier to conduct research that was not explicitly about race. For instance, one scholar described such research as "a lot less nerve wracking. It's super easy. I mean from start to finish… It doesn't mean anything if it doesn't work. Whereas this whole [race-related paper], I feel like I have something to prove when it doesn't work." (RM_4). Finally, another scholar reported feeling less like they needed to "perform" during non-race related talks:

> "Those presentations are usually lighter. I can tell more jokes, and it's more back and forth with the audience … They don't have a sense of heaviness. I'm able to perform

> the talk in a more lighthearted way, and therefore, I don't necessarily feel drained. I just feel like, 'Okay, that was cool.'" (RM_12)

When probed about potential racial differences presenting racial scholarship, White scholars often described their Whiteness as protective; their identity deterred harsh criticism and allowed them to present with less pushback. As one White scholar pondered, "Being a White male I'm able to use language that might be read as accusatory … language around privilege and anti-racism. And [this language], people have said this to me, can be read as 'accusatory' and make people uncomfortable." (WH_15). This scholar also noted a difference in risk for White scholars versus scholars of color when presenting racial scholarship:

> "[The work] might have serious implications [for scholars of color] in terms of, 'I'm going to be harassed,' or 'I'm going to receive really nasty emails,' or 'Someone's going to tell me that I don't belong in this space,' or 'I might not get a job offer.' … By virtue of being a White man … it just makes it easier for me." (WH_15)

White scholars expressed that they felt they were seen as "more objective"(WH_4), given more "credibility" (WH_4), and seen as less invested, making them "much less threatening because it doesn't look like I have skin in the game" (WH_18), due to their Whiteness. As one scholar described: "I've been really lucky, because, in academic settings … I don't think I've ever encountered anyone who pushed back in kind of a really personally negative way against the research or felt like they were being attacked in any sense." (WH_20). Other White scholars similarly described feeling "lucky" that they did not receive harsh criticism of their racial scholarship: "No one's ever tried to overtly challenge me in any way about the stuff I'm concluding"(WH_11).

Additional examples of constraint. Scholars received feedback from mentors to "water down your work and say that it applies to everybody… or say it's going to be about

diversity" (RM_15). This was often framed as protective: "They're attempting to look out for me and say like, 'Look, like we want you to produce high quality work, and do well, and get a job, and so on.'" (RM_2). This advice was explicitly given to assuage the discomforts of a White audience:

> "The biggest trope I get is 'Don't scare these White people. Don't scare these White people. You got to talk them out of this thing. You got to talk about it this way. You can't say that. You can't say this.' And I'm like, 'No, but what I'm saying is the truth.' They're like, 'Yeah, but you have to make sure they hear you.'" (RM_7)

Constraint was described as key to being seen as professional, even when that meant subduing the truth: "When like minoritized scholars hedge … the idea is that you're a better scientist. … The moment that you say more out there things, you are now like more of an activist. … It's just deemed to be 'more professional'" (RM_12). For scholars of color, "professional" meant being a calm, detached, and objective scientist.

As with objectivity interrogations, constraint was often employed for the benefit of White scholars and was explicitly informed by scholars' racial identities:

> "I also have to handhold people through some things that are relatively uncomfortable and do so in a way that is friendly. To be threatening in a friendly way, I think that is informed by the fact that I show up as a [racial identity redacted] to these rooms." (RM_18)

This meant that scholars often mentioned practicing self-interrogation, to remind themselves to remain silent rather than upset White colleagues: "If I thought something was ignorant I had to be like, do I say something or do I keep quiet?... If I get upset by something it's like, 'oh there goes the spicy feisty [racial identity redacted]'" (RM_5), and "It makes me probably more hesitant if anything for what I do…and I think I'm processing the identity politics of it in a personal way of like, 'Oh, is that going to bother someone if I say that?'" (RM_24).

Additional examples of avoidance. Patterns of avoidance meant, for some scholars, that they ended up studying topics that were not of particular interest to them.

> "I just ended up doing what he was doing and kind of like dropped my interests. … He was doing work on confrontation and it was basically looking at how Black folks can ease tensions within interracial interactions…Early on, I did want to do a lot of interracial work and Black folks' perceptions of other Black folks. But … like no one is going to care about this particular interest." (RM_9)

Additional examples of overpreparation. Scholars of color also overprepared to eschew negative stereotypes based on these scholars' racial identities and avoid potential interrogations. One scholar described this as codeswitching, saying "I'm always over-prepared with my work. I think I've been trained to do that, to try to avoid stereotypes, which is a form of code-switching" (RM_17). Another stressed the importance of establishing competence early, noting "In the literature review part, and I think that helps, when you flagrantly demonstrate competence, right? I think on the front end it might have deterred people from engaging in unhelpful competence questioning behaviors." (RM_3).

These overpreparation efforts were not always successful in answering questions of objectivity. As one scholar presenting on stereotypes noted:

> "There's literally books and books and journal articles and chapters and entire issues of work in this space…I was like just trying to explain to him how all of this works, how structural racism and inequality works, how it leads to stereotyping. And he just totally was not buying it. He's like, 'I just don't think it's real.'" (RM_13)

Additional examples of quantification. Although it required more time and effort to gain additional expertise, scholars of color perceived quantification as a protective measure to prevent criticism of their research:

> "I'm going to do clean, strong work and they can't challenge that part. They can say 'I disagree.' They can say 'I don't really like it,' but they can't say 'Your methods were flawed' or 'You didn't have a big enough sample,' or 'The analyses were suspect,' or 'You didn't preregister.' None of that." (RM_23)

Importantly, quantification often occurred with toning down. Additional quantification allowed scholars of color to be more measured when presenting findings, reporting that they "let the data speak for itself" (RM_7), and "try not to speak beyond my data" (RM_17).

Additional examples of exacting communication. Another scholar mentioned that exacting communication was a strategy employed in order to avoid offending majority group members: "I have to be a lot more careful with my words to not offend majority group members that feel left out … within presenting there's just more consciousness of my words when I'm talking about sensitive topics." (RM_11).

Additional examples of objectivity armoring among White scholars. . In our interviews, we observed less exacting communication patterns employed by White scholars:

> "I threw that presentation together the week that I was giving it. … I wasn't exactly taking a big step back and thinking about the way that it would come across to everybody or the broad themes that I was emphasizing. It was more just like I was like, 'Okay, I have a lot of cool data.'" (WH_11)

This contrasts with the intense overpreparation exhibited by scholars of color. Indeed, another White scholar reported that they "try not to overprepare" (WH_18).

We also provide negative cases, wherein White scholars do report objectivity interrogations or self-presentational shifts. In the rare cases when White scholars did report objectivity interrogations, they were not described as particularly concerning:

> "There's been negative reactions there, and you look at the source of it and it's who you would expect. It's the people who, in my mind, are causing some of the biggest problems in society and they should be threatened by this work identifying the source of their threat. … It doesn't really bother me. If anything it encourages me, or strengthens my resolve." (WH_12)

This reflection underscores the relative positions that White scholars and scholars of color hold; rather than discouraging this White scholar, interrogations seemed to encourage them to continue their work. Another scholar reported that they "[weren't] too affected" by interrogations, describing them as "weird question[s] … something that a lot of race

researchers get and especially researchers of color" (WH_5). Another reported that an interrogation they received was not "specific necessarily to that talk" suggesting that "it might just be the audience at [conference]" (WH_9). When White scholars did report self-presentational shifts, they only did so to attend to specific methodological context:

> "I'm mostly worried about, 'Oh are people going to be okay with how I preprocessed this data?' Or 'Oh, is this computational approach actually legitimate?' That's why I really haven't thought at all about, 'Oh, how are people going to feel about my presenting intergroup work?'" (WH_3)

Generally, White scholars were much less likely to receive interrogations to their work. When they did, they were often less affected by it and less likely to shift their self-presentational strategies in response. One White scholar reflected on how their identity protected them from interrogations around racial scholarship: "Language around privilege and anti-racism...can be read as 'accusatory' and make people uncomfortable. And in my head I think I'm better able or it's safer for me." (WH_15).

Some White scholars recognized their own inability to truly understand the experiences of scholars of color conducting racial scholarship: "Because I'm at a distance, I think it's hard for me to truly understand what that experience is like to listen to [interrogations] as a member of the target group." (WH_9).

Results: Archival Analysis

Full model results for our linear regression analysis predicting power word use (Table S1), positive emotion word use (Table S2), and negative emotion word use (Table S3) are shown below.

Table S1. Linear regression of power language

	Beta	t	Sig.

Intercept		-2.856	.004
Racial identity	-.102	-3.497	.000
Impact factor	.017	.559	.576
H-index	-.016	-.511	.609
Publication year	-.073	-2.215	.027
Word count	.106	2.976	.003

Table S2. Linear regression of positive emotion language

	Beta	t	Sig.
Intercept		-3.890	.000
Racial identity	.101	3.485	.001
Impact factor	-.036	-1.180	.238
H-index	.090	2.871	.004
Publication year	-.044	-1.336	.182
Word count	.141	4.003	.000

Table S3. Linear regression of negative emotion language

	Beta	t	Sig.
Intercept		-.826	.409
Racial identity	-.031	-1.061	.289
Impact factor	-.017	-.561	.575
H-index	.001	.018	.986
Publication year	.014	.430	.667
Word count	.032	.908	.364

Full model results for our linear mixed model analysis with abstracts nested within journals appear below. The analysis in Table S4 shows our analysis for power word use, Table S5 for positive emotion word use, and Table S6 for negative emotion word use.

Table S4. Linear mixed model analysis of power language

	Estimate	Std. Error	df	t	Sig.
Intercept	-92.958058	32.338796	1013.852	-2.875	0.004
Racial identity	-0.225941	0.092848	1180.451	-2.433	0.015

Impact factor	-0.022643	0.031975	23.003	-0.708	0.486
H-index	-0.002640	0.003361	1157.888	-0.785	0.432
Publication year	0.048185	0.016107	1009.459	2.992	0.003
Word count	-0.002244	0.002169	1034.886	-1.035	0.301

Table S5. Linear mixed model analysis of positive language

	Estimate	Std. Error	df	t	Sig.
Intercept	-79.390305	21.099374	782.652	-3.763	0.000
Racial identity	0.226256	0.061694	1082.186	3.667	0.000
Impact factor	-0.016020	0.016278	45.286	-0.984	0.330
H-index	0.006328	0.002233	1158.651	2.833	0.005
Publication year	0.040716	0.010509	777.615	3.874	0.000
Word count	-0.001397	0.001413	654.037	-0.988	0.323

Table S6. Linear mixed model analysis for negative language

	Estimate	Std. Error	df	t	Sig.
Intercept	-15.295743	26.189703	979.549	-0.584	0.559
Racial identity	-0.102656	0.075573	1173.303	-1.358	0.175
Impact factor	0.006911	0.023763	32.192	0.291	0.773
H-index	0.000158	0.002733	1158.414	0.058	0.954
Publication year	0.008513	0.013044	975.312	0.653	0.514
Word count	0.001103	0.001757	989.947	0.628	0.530

Although the modal number of abstracts authored by each lead author is one in our data, a subset of authors appears in our data with more than one abstract. To account for the outsized impact of these authors in our data we randomly removed any additional abstracts authored by each lead author and re-ran our independent samples t-test comparing word use for power, positive emotion, and negative emotion words. The results were consistent with those reported in the manuscript. As in our analysis reported in the manuscript, lead authors of color used fewer power related words (M = 3.06) than White authors (M = 3.61; t(1295) = 3.104, p = .002), and more

positive emotion (*M* = 2.43) words than White authors (*M* = 2.16; $t(1295) = -2.366$, $p = .018$). Again, negative emotion word usage did not differ by lead author race $t(1295) = -0.277$, $p = .782$.

We also examined language use as a function of authorship team diversity. One expectation is that the dynamics of language use associated with lead author race would also be similar for more racially diverse authorship teams. We created an index, in this regard, with diversity measured in terms of the proportion of authors who were scholars of color on an authorship team. Scores of 0 indicate a team is all White and scores of 1 indicate a team includes only scholars of color (*M*=0.26, *SD* = 0.31). The diversity index correlated with lead author diversity $r(1688) = .753$, $p < .001$ indicating that lead authors of color tended to author papers on more diverse teams. Moreover the diversity index was associated with reduced power language $r(1688) = -.072$, $p = .003$, and more positive language $r(1688) = .077$, $p = .002$ in the same direction as lead author race. This exploratory analysis lends additional data to the idea that authorship team diversity relates to language use in a similar pattern to how lead author race does.
